# A Clustering Study of Dietary Patterns and Physical Activity among Workers of the Uruguayan State Electrical Company

**DOI:** 10.3390/nu16020304

**Published:** 2024-01-19

**Authors:** Maria Mercedes Medina-Vadora, Julio Plaza-Diaz, Francisco Jesús Llorente-Cantarero, Cecilia Severi, Carlos Lecot, María Dolores Ruiz-López, Ángel Gil

**Affiliations:** 1 Department of Nutrition and Food Sciences, Faculty of Pharmacy, University of Granada, 18071 Granada, Spain; mechemv@gmail.com (M.M.M.-V.); mdruiz@ugr.es (M.D.R.-L.); 2 Department of Biochemistry and Molecular Biology II, University of Granada, 18071 Granada, Spain; jrplaza@ugr.es; 3 Instituto de Investigación Biosanitaria ibs.GRANADA, Complejo Hospitalario Universitario de Granada, 18014 Granada, Spain; 4 Children’s Hospital of Eastern Ontario Research Institute, Ottawa, ON K1H 8L1, Canada; 5 Department of Specific Didactics, Faculty of Education, Maimónides Institute of Biomedicine Research of Córdoba (IMIBIC), University of Córdoba, 14071 Córdoba, Spain; fllorente@uco.es; 6 CIBEROBN (Physiopathology of Obesity and Nutrition), Instituto de Salud Carlos III (ISCIII), 28029 Madrid, Spain; 7 Department of Preventive Medicine, School of Medicine, Universidad de la República Oriental del Uruguay (UdelaR), Montevideo 11800, Uruguay; severi.cecilia@gmail.com; 8 Uruguayan Society of Collective Health (SUSAC), Montevideo 11800, Uruguay; 9 Department of Occupational Health, National Administration of Power Plants and Electric Transmissions (UTE), Montevideo 11800, Uruguay; clecot@ute.com.uy; 10 Biomedical Research Center, Institute of Nutrition and Food Technology “José Mataix”, University of Granada, 18016 Granada, Spain; 11 Iberoamerican Nutrition Foundation (FINUT), 18016 Armilla, Spain

**Keywords:** cluster analysis, dietary patterns, food groups, principal component analysis, physical activity, sedentary behavior

## Abstract

Recent studies have shown that certain nutrients, specific food groups, or general dietary patterns (DPs) can promote health and prevent noncommunicable chronic diseases (NCCDs). Both developed and developing countries experience a high prevalence of NCCDs due to poor lifestyle habits, DPs, and low physical activity levels. This study aims to examine the dietary, physical activity, sociodemographic, and lifestyle patterns of Uruguayan State Electrical Company workers (the IN-UTE study). A total of 2194 workers participated in the study, providing information about their sociodemographics, lifestyles, and dietary habits through different questionnaires. To identify DPs from 16 food groups, principal component analysis (PCA) was performed. A hierarchical cluster algorithm was used to combine food groups and sociodemographic/lifestyle variables. Four DPs were extracted from the data; the first DP was related to the intake of energy-dense foods, the second DP to the characteristics of the job, the third DP to a Mediterranean-style diet, and the fourth DP to age and body mass index. In addition, cluster analysis involving a larger number of lifestyle variables produced similar results to the PCA. Lifestyle and sociodemographic factors, including night work, working outside, and moderate and intense PA, were significantly correlated with the dietary clusters, suggesting that working conditions, socioeconomic status, and PA may play an important role in determining DPs to some extent. Accordingly, these findings should be used to design lifestyle interventions to reverse the appearance of unhealthy DPs in the UTE population.

## 1. Introduction

In both developing and developed countries, poor dietary habits, low physical activity (PA), sedentarism, frequent alcohol consumption, and tobacco use are associated with an increased mortality rate and prevalence of noncommunicable chronic diseases (NCCDs) [1]. Following the World Health Organization (WHO), NCCDs include cardiovascular diseases (CVDs) (myocardial infarctions and strokes), cancer, chronic respiratory diseases (chronic obstructive pulmonary disease and asthma), and diabetes [2]. In 2019, a 30-year-old living in the Region of the Americas had a 14.0% chance of dying from one of the four major NCCDs before the age of 70. A high of 31.3% was recorded in Haiti, while a low of 9.5% was recorded in Costa Rica. There is a 16.5% mortality rate from NCCDs in Uruguay [3].

It has been shown that NCCDs are primarily caused by four specific behaviors (tobacco use, inactivity, unhealthy diets, and alcohol abuse) that lead to four critical metabolic/physiological changes (overweight and obesity, high blood pressure, and elevated blood glucose and cholesterol) [2]. Obesity has reached epidemic proportions worldwide, and in 2021, it was responsible for 2.8 million NCCD deaths in the Americas [4]. Overweight and obesity have tripled in the region over the past 50 years and are currently affecting 62.5% of the population (64.1% of men and 60.9% of women) [5]. The Pan American Health Organization estimates that obesity affects 28 percent of the adult population (26 percent of men and 31 percent of women) [6]. Globally, unhealthy diets, primarily sugar-sweetened beverages, and saturated fats have been linked to an increased risk of CVDs [7]. In adults, NCCDs are associated with behavioral risk factors developed in adolescence, and two-thirds of premature deaths are caused by behavior adopted during adolescence [8,9].

Many countries, including Australia and Canada, have reformed their national strategies to prevent NCCD risk factors by emphasizing the importance of PA, sleep, and proper nutrition during childhood and adolescence [10,11].

Traditional studies of food consumption have focused on the ingredients and nutrients contained in food. In recent years, other approaches have been developed, because diet is multidimensional and complex [12,13]. Various factors influence the choices we make as individuals, as a society, and as a culture [14].

Dietary patterns (DPs) have been used since the early 1980s to synthesize multiple related dietary components into variables representing key dietary habits and the overall diet of free-living individuals. There is also a strong interest in DPs due to the well-known interactive effects of foods eaten together, as well as data dimensionality and multiple testing issues affecting the statistical analysis of many single food groups or nutrients [15].

Empirical DPs and PA, together with other covariates, e.g., socioeconomic and lifestyle factors, have been derived using principal component analysis (PCA), exploratory factor analysis, or cluster analysis [16].

PA is defined as any movement produced by skeletal muscles that requires the expenditure of energy [17]. The activities described above are considered sustainable physical activities, which are performed with sufficient duration, frequency, and intensity to promote good health [17,18]. PA at the community level is considered to be the first indicator of health by the WHO [19]. Regular PA can reduce the risk of NCCDs, including cancer, CVDs, and type 2 diabetes [20,21,22].

During the 2nd International Conference on Nutrition in 2017, the United Nations Food and Agriculture Organization (FAO) recognized that some socioeconomic and environmental changes may affect eating habits and PA [23]. As a result of increased sedentary habits and increased consumption of food products with a high fat content, particularly saturated fats and trans fats, sugars, and salt or sodium, we are becoming increasingly susceptible to obesity and NCCDs [24].

This phenomenon is nothing new for Uruguayan society, and in 2013 the Second National Survey of Risk Factors for Noncommunicable Diseases was conducted. The main findings indicate that 64.9% of people aged 25 to 64 are overweight or obese. Comparing this value to the 56.6 percent obtained from the First National Survey of Risk Factors for NCCDs in 2006, there is a statistically significant difference, resulting in an 8.3 percentage point increase [25].

As reported by the International Labor Organization (ILO), workers’ health and productivity are negatively impacted by poor workplace nutrition [26,27]. According to this study, workplaces can be used as a platform for developing nutritional interventions that improve health. In many cases, long trips to and from worker homes to workplaces can be added to this statement, since workers spend half or more of their time at work [28].

The unbalanced intake of food during work can reduce productivity by up to 20% and result in significant health problems. According to Branca et al. [29], businesses can reduce the burden of NCCDs (CVDs and tumors) on the general population by promoting healthy eating among their employees [29].

According to Uruguayan government guidelines for PA, active behaviors should be included in daily life from an early age, screens should be limited, and quality sleep should be promoted to promote healthy lifestyles that will continue into adolescence, youth, and adulthood and be passed on to future generations [30].

In Uruguay, malnutrition problems caused by excess prevail at all ages due to an advanced stage of the nutrition transition [31]. Approximately 60% of all health care costs in Uruguay are attributed to NCCDs, and 70% of all deaths are due to them [32,33]. Cardiovascular diseases are the leading cause of death in adults of working age, with serious consequences for their families, societies, and organizations [34]. Finally, the ANIBES, [35,36], PREDIMED [37], and CORDIOPREV [38] studies, as well as a study in Uruguay [39], published similar reports. In addition, dietary approaches to stop hypertension (DASH) diets emphasize fruits, vegetables, whole grains, lean proteins, and low-fat dairy products [40,41]. This DP limits foods with added sugar and foods high in saturated fat, such as fatty meats, full-fat dairy products, and tropical oils [42]. One of the most important aspects of the DASH diet is that sodium is capped at 2300 milligrams daily, which is usually reduced to about 1500 milligrams by followers [43,44].

The present study aimed to analyze the dietary, PA, sociodemographic, and lifestyle patterns among the workers of the Uruguayan State Electrical Company as part of the IN-UTE (Nutritional Research at the National Administration of Power Plants and Electric Transmissions UTE, Uruguay) study.

## 2. Materials and Methods

### 2.1. Study Design and Samples

The data used in this manuscript were obtained as part of the IN-UTE study, which was a transversal observational study addressed to workers who began working at UTE between January 2010 and December 2017. A detailed description of the IN-UTE study design, protocol, and methodology can be found elsewhere [45]. In brief, the IN-UTE study evaluated the dietary and nutritional intake, DPs, and PA of UTE workers [45].

Two thousand one hundred and ninety-four workers (1393 men and 801 women) of UTE participated in the study and were interviewed face-to-face. The IN-UTE study was conducted following the Declaration of Helsinki, and the Research Ethics Committee of the University of Granada (Spain) reviewed and accepted the final protocol according to the ethical standards of the Declaration of Helsinki of 1964. The document is coded as 1088/CEIH/2020, dated 3 March 2020. In a subsequent step, the study was registered at ClinicalTrials.gov (Protocol Registration and Results System-PRS-Receipt Release Date: NCT04509908).

Participants were informed about the study before any information was requested. If a participant did not provide a signed consent form or did not complete any study phase, they were excluded from the study. Participants were able to stop the interviews at any time, and their previously collected partial information was excluded from any analysis in these circumstances. Upon obtaining the medical histories and surveys, the data were anonymized.

#### 2.1.1. Dietary Survey and Data Collection

We used a food frequency questionnaire (FFQ) that had previously been modified, adapted, and validated according to the ANIBES [35,46] study based on the adult population and eating habits of Uruguayans [25,47]. There are 159 items on this FFQ form, which was sent via the web to employees who meet the criteria. It takes approximately 30 min to complete this form.

The STEPS form was used as a reference (progressive method of PAHO for the surveillance of chronic disease risk factors (PAHO-STEPS)) [25]. The adaptation was validated for the study population [25].

Participants were asked a series of quantitative questions regarding their usual food consumption over the past 12 months, organized by 10 food groups, including 159 different foods, including those most commonly consumed by Uruguayans [48,49,50]. Due to the wide variety of food products available today, these 10 groups were further divided into 16 groups.

In this study, the following food groups were considered: “milk and dairy products”, “eggs”, “meat”, “meat products”, “cereals”, “fruits”, “vegetables”, “tubers”, “legumes”, “nuts”, “ready to cook” (industrial precooked foods prepared with the expectation of being heated/cooked in the following ways: frying, microwave, oven, stovetop—e.g., frozen pizza, frozen vegetables, etc., or baking) divided into “ultra-processed salted”, “ultra-processed sweetened”, “oils and fats”, “beverages”, “fish and fish byproducts”, and “added salt” [45].

The energy and nutrient intakes were estimated using EvalFINUT 2.0 software [51]. The food database used was the National Nutrient Database for Standard Reference (USDA) [52], which also included Uruguayan foods and preparations.

#### 2.1.2. Physical Activity and Sedentary Behavior Questionnaire

Participants’ daily PA was evaluated following the WHO recommendations for PA, sedentary behavior, and sleep for adults between the ages of 18 and 64 [53].

The Self-Questionnaire International Physical Activity Questionnaire (IPAQ) was used in this study to estimate energy expenditure (EE). Briefly, IPAQ is a standardized measure of habitual PA practiced by populations in different countries and sociocultural contexts, which involves a 7-day recall of PA [54,55].

Based on the MET value from the Youth Compendium, the basal metabolic rate (BMR) measured or computed, and the duration of each activity, the energy cost is computed as follows:

An adult’s BMR is predicted by using the Schofield equation with the following formula: METs × BMR (kcal/min) × duration (min) [56].

PA was measured based on the intensity of the activities and classified according to the latest recommendations of WHO guidelines [57,58] as sedentary (≤1.5 METs), light-intensity activities (1.5–4 METs), moderate-intensity activities (4.1–7 METs), and vigorous-intensity activities (>7 METs) [57].

#### 2.1.3. Anthropometric Data

The anthropometric parameters (weight, height) of the participants were collected by auto report.

#### 2.1.4. Covariates

Medical doctors specializing in occupational medicine collected the medical history and socioeconomic data and wrote the records, which were later digitized. Sociodemographic data included age, sex, marital status, occupation, place of residence, and educational level.

Characteristics and risks of the job included the organization and division of tasks (rotating rests, rotating shifts, night shift, on-call, customer service), the risk of work accidents, nonwork accidents, prolonged absenteeism, and the reasons for it [45].

### 2.2. Data Analysis

#### 2.2.1. General

The statistical analyses were conducted using IBM SPSS Statistics for Windows, version 25.0 (IBM Corp., Armonk, NY, USA) and R version 3.6.1 (R Foundation for Statistical Computing, Vienna, Austria). For each variable, descriptive statistics were calculated. Except where otherwise stated, all results are expressed as the mean ± standard deviation. Sociodemographic and job variables were compared using the chi-squared test.

#### 2.2.2. Dietary and Physical Activity Patterns

To identify the underlying DPs, PCA was performed with several input variables, including average weight consumed by individuals from 13 food groups (g/day), job-related variables (cumulative risk factors, work outside the apartment, 24 h on-call duty), PA variables (vigorous and moderate PA), body mass index (BMI), and age [59]. As part of the multicollinearity evaluation, the R-matrix Bartlett’s test of sphericity was examined, as well as the Kaiser–Meyer–Olkin (KMO) measure of sampling adequacy. To determine the degree of intercorrelation between variables, we used a KMO value greater than 0.60. To enhance interpretability, we orthogonally rotated the factors (the Varimax option).

We retained factors that met the following criteria: factor eigenvalue > 1.2, identification of a breakpoint in the scree plot, proportion of variance explained, and interpretability of the factors [16]. A factor loading matrix was used to describe the strength and direction of the associations between patterns and food groups. Patterns identified were based on food groups with factor loadings greater than 0.20 and communality greater than 0.20. Factor scores were calculated by summing the observed intake of the component food items weighted by the factor loading. In general, a high factor score indicates a high intake of the foods constituting a given food factor, while a low factor score indicates a low intake of those foods or level of PA or low job characteristics. Data from input variables were displayed as a two-dimensional radar chart based on axes that started from the same point to display multivariate data.

#### 2.2.3. Lifestyle Patterns with Clustering Analysis

Cluster analysis was employed as an exploratory tool to uncover natural clusters within the dataset that would not otherwise be evident and to automatically determine the “best” number of clusters using SPSS v.25 (IBM, Chicago, IL, USA). After that, unsupervised hierarchical clustering analysis was performed using the R v.3.6.3 package on the FFQ and lifestyle variables (anthropometric measure, sociodemographic and job variables) to develop clusters of subjects with similar characteristics (R package heatmap). As a linkage criterion, Ward’s method was used to group the clusters in the distance matrix based on Euclidean distances. Based on the distance matrix, the clustering algorithm identifies the closest observations (e.g., subjects with similar dietary and lifestyle behaviors, in rows) and merges them within the same cluster until all clusters have been merged. On the basis of the silhouette method (R package Nbclust), three clusters were retained among subjects and lifestyle/dietary variables. According to the Agnes function in the R package cluster, the agglomerative coefficient was always greater than 0.85 [60].

## 3. Results

### 3.1. Population Characteristics

Table 1 provides a general description of the working population at UTE. The majority of the population was male (63.5%), with an average age of approximately 41 years. Approximately 35.7% of the population had a normal BMI, and interestingly 63.4% were classified as overweight (41.0%) and obese (21.6%). There was a significant difference in the distribution of BMI categories according to sex. The variables of the job revealed that 37.4% of the population had at least one risk factor, whereas travel to other departments, rotation schedules, and being on call 24 h a day contributed 17.7%, 11.1%, and 12.4%, respectively (Table 1).

According to Table 2, more than 10% of employees ate two or fewer meals per day, while more than 55.6% had four meals per day. Among the main daily servings were beverages, cereals, fruits, and natural juices, as well as meat and meat products. Table 2 shows the international dietary recommendations based on the U.S. Dietary Guidelines [61,62] to compare with the actual intakes of different types of foods in the considered population. Compared to U.S. dietary recommendations, Uruguayans consume fewer fruits, vegetables, cereals, nuts, fish and fish byproducts, oils and fats, eggs, and milk and dairy products. The consumption per gram/day of meat and meat products was higher than that of the U.S. dietary guidelines. The sleep and PA patterns are shown in Table 3. The majority of the population (83.8%) slept six to eight hours per night. It is important to note that in the distribution of gender men accounted for 85.4% of those who slept from 6 to 8 h, whereas women accounted for only 81.0% (Table 3). In terms of PA levels, no differences were observed between men and women. In both gender groups, vigorous PA accounted for the greatest amount of PA in terms of METs/week (Table 3).

Based on the categorization of PA according to the number of hours performed, Table 4 shows that there are differences between men and women in terms of moderate and vigorous PA, as well as sitting time. There was a lower level of moderate and vigorous PA among women than among men; indeed, the number of hours spent sitting over 3 h was higher in the female population (Table 4).

### 3.2. Dietary Patterns

**Figure 1:**
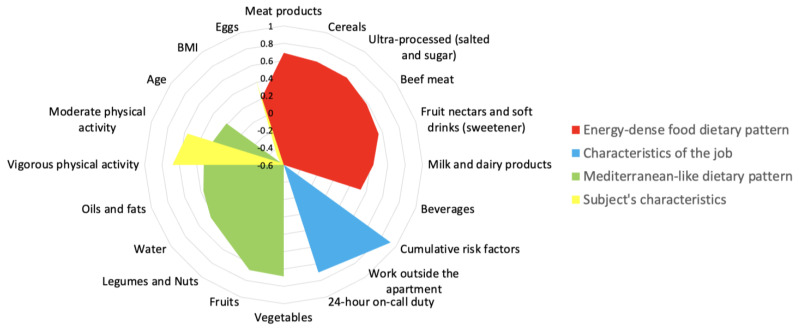
Dietary patterns extracted from the principal component analysis of input variables in the IN-UTE study (n = 2194).

DPs were calculated using the PCA statistical method. In the UTE population, Bartlett’s tests of sphericity and KMO supported the appropriateness of factor analysis. In the analysis, Bartlett’s tests of sphericity were significant, and the KMO value was greater than 0.633. Based on the input variables, as mentioned above, four major factors were identified through PCA, accounting for 40.68% of the variance (Table S1). As shown in Figure 1, these components are represented as DPs for the entire population.

We observed four major factors or components in UTE workers: The first component (energy-dense foods) had high positive loadings on meat products, cereals, ultra-processed foods (salted and sugared), beef meat, fruit nectars and soft drinks with sweeteners, milk and dairy products, and beverages. A second component (characteristics of the job) was characterized by high positive loadings on cumulative risk factors, work outside the apartment, and 24 h on-call duty. In the third component (Mediterranean-like diet and lifestyle), vegetables, fruits, legumes and nuts, oils and fats, eggs, and variables of PA (moderate and vigorous PA), were associated with high positive loadings. Last, the final component (subjects’ characteristics) was associated with positive loadings for moderate and vigorous PA levels and negative loadings for BMI and age (Figure 1).

### 3.3. Dietary and Lifestyle Patterns

**Figure 2:**
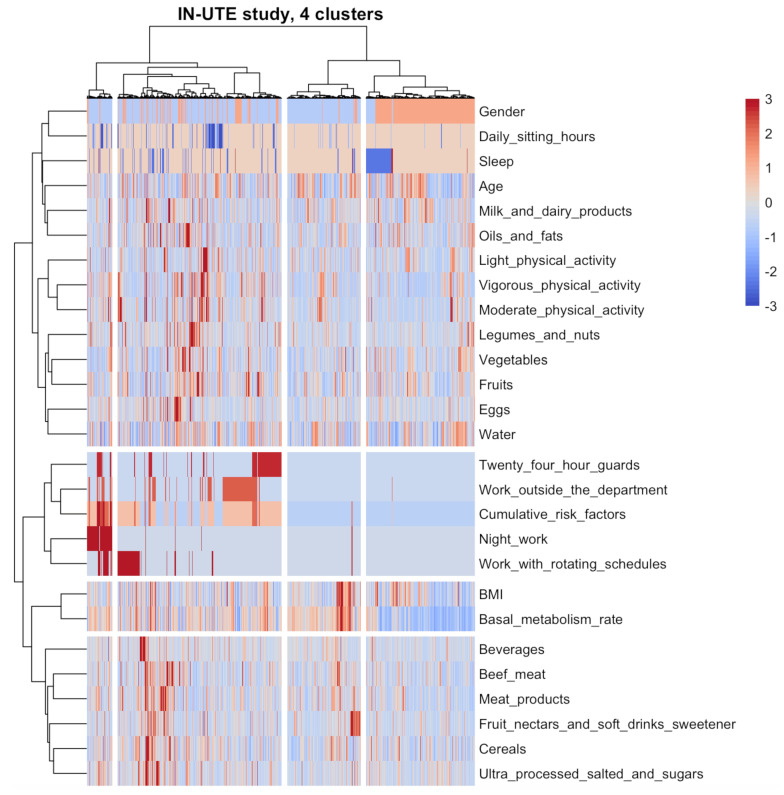
Clusters of subjects and dietary/lifestyle variables were identified via hierarchical clustering in the IN-UTE study (n = 2194). The clusters are visually separated by longitudinal marks on vertical and horizontal faces (clusters of subjects or dietary/lifestyle variables, respectively). The vertical and horizontal dendrograms denote the relationship between the clusters, i.e., similar observations. The color bar refers to levels above (red) or below (blue) the mean intake of dietary variables or mean scores of lifestyle variables. Increased color intensities indicate larger differences around the mean. Abbreviations: BMI: body mass index.

Figure 2 illustrates the clusters of subjects within the UTE workers’ study sample based on their dietary and lifestyle characteristics. The clustering algorithm identified four main clusters of subjects and dietary/lifestyle variables (the silhouette method suggested four clusters).

We found four clusters of subjects and dietary/lifestyle variables in the IN-UTE study (Figure 2). According to the dietary/lifestyle clusters (in rows), cluster 1 possessed a Mediterranean-like diet and variables related to PA; cluster 2 consisted primarily of job variables (24 h guards, night work, rotating schedules, working outside the department, cumulative risk factors); cluster 3 included BMI and basal metabolic rate; and cluster 4 included energy-dense foods.

Considering the characteristics of the subjects (by columns), the first cluster is composed of younger men who consumed fewer food groups and had higher night work and cumulative risk factors in their jobs. Low levels of moderate and vigorous PA were observed in these subjects, as well as a low intake of beef and meat products. Second, there is a major cluster of men of average age who regularly consume a Mediterranean-like diet. Additionally, these subjects work more at night and consume energy-dense products (beef and meat products). This third cluster is predominantly composed of men who consumed on average Mediterranean diet products and consumed higher amounts of energy-dense foods. These subjects work less at night and schedule rotations, and it appears that this group has the highest BMI values. Furthermore, the fourth cluster is largely composed of women, with a high value for PA and Mediterranean-style diet. These women in general have lower levels of activities related to night and a schedule of rotations and consume less energy-dense products (Figure 2).

## 4. Discussion

The major findings of the present study relate to the subjects and job characteristics of UTE workers and their associations with specific DP and PA levels. The majority of people are male (63%), and the average age is 41. Approximately 35.7% of the population had a normal BMI, and interestingly 63.4% were overweight (41.8%) and obese (21.6%). BMI categories were distributed differently by gender. In addition, 37.5% of the population had at least one risk factor, while traveling to other departments, rotating schedules, and being on call 24 h a day accounted for 17.1%, 11.0%, and 12.1%, respectively.

It is estimated that the prevalence of overweight and obesity in Uruguay increased from 57 to 65%, hypertension increased from 30 to 39%, and diabetes increased from 5 to 8% between 2006 and 2013 [63]. Additionally, 30 percent of the adult population is physically inactive [63]. It is estimated that the daily energy consumption of Uruguayan adults (2432 kcal) exceeds the daily calorie requirement by 19% [64], approximately 90% of adults consume insufficient amounts of vegetables and fruits [63], salt consumption is high [65], and highly processed foods have increased significantly in consumption. As a result, the dietary habits of Uruguayans may pose a threat to their health [39]. In our study, the majority of employees consumed four meals per day, compared to over 10% who consumed two meals or fewer per day. Beverages, cereals, fruits, natural juices, and meat and meat products were among the most common daily servings. However, the Uruguayan diet is lower in fruits, vegetables, cereals, nuts, fish and fish byproducts, fats and oils, eggs, and milk and dairy products than the U.S. diet. Meat and meat products were consumed at a higher rate than that recommended by the U.S. dietary guidelines.

Seventy-four percent of USA adults are overweight or obese, and six out of ten adults suffer from one or more diet-related chronic disease, such as cardiovascular disease, type 2 diabetes, or obesity. Since 1980, the U.S. Departments of Agriculture and Health and Human Services have published Dietary Guidelines for Americans [61]. Overweight and obesity are also frequent in Latin American countries [66]. In Uruguay, 64.9% of adults aged 25 to 64 are overweight or obese, according to a national survey conducted in 2013 [25].

In recent years, there has been an increase in research supporting the idea that certain types of nutrients, specific food groups, or overarching DPs are beneficial to health and contribute to the prevention of NCCDs [67]. In contrast, bad lifestyle habits, including DPs and low PA, contribute to the increase in the prevalence of NCCDs in both developed and developing countries [2].

Global public health is increasingly concerned with sleep problems because poor sleep leads to impaired motivation, emotional well-being, and cognitive function, as well as an increased risk for NCCDs and all-cause mortality, even when the symptoms do not meet the threshold for clinical sleep disorders [68,69,70]. Approximately 83.8% of the population in our study slept six to eight hours per night. The gender distribution indicated that 85.4% of men slept between 6 and 8 h, whereas 81.0% of women slept between 6 and 8 h.

Individuals exhibit DPs over time, such as their regular eating habits [67]. The DPs represent the combination of foods recommended for healthy living or the foods and beverages they consume over some time. To help everyone, regardless of age, race, ethnicity, or current health status, a healthy DP must take into account personal preferences, cultural traditions, and budgetary considerations [61].

Several decisions need to be made regarding the format and potential transformation of the input variables in these analyses, the number of input variables and food grouping schemes, the estimation method, and the criteria for selecting the model, including how to select the number of DPs to retain [71]. The underlying eating patterns can be revealed through either PCA or cluster analysis despite clear differences in approaches and interpretations [13], and most identified DPs generate valid dietary constructs indicating the dietary characteristics of the populations considered [14].

In our study using PCA, all variables were first measured to extract four components. The first component was related to energy-dense foods, the second component was related to the characteristics of the job, the third component was related to a Mediterranean-like diet and lifestyle, and the fourth was essentially related to age and BMI. Finally, cluster analysis using a broader number of lifestyle variables produced similar results as PCA.

Other authors have reported similar results regarding DPs in Uruguay based on a geographically defined sample of adults in Montevideo identifying three DPs: Meat, Prudent, and Cereal and Mate in a small sample (n = 294) [39]. They were able to define a DP for almost 300 participants in which meat was present. Similarly, our first component from PCA shows meat products, cereals, ultra-processed foods (salted and sugared), beef meat, fruit nectars, soft drinks with sweeteners, milk, and dairy products.

In the case of Spain, the ANIBES study reported four DPs using factor analysis, one of which resembled the traditional Mediterranean diet and lifestyle. There were two distinct groups of DPs, PA behaviors, and sedentary behavior in Spanish children and adolescents on weekdays. There was a high proportion of girls with a low level of PA and poor diet, as well as a higher level of PA, low sedentary behavior, longer sleep duration, and a healthier diet lifestyle pattern [36]. Our study found that most UTE workers consumed a high amount of energy-dense foods and had low levels of PA, which could negatively affect their health.

As shown in the IN-UTE study, some UTE workers exhibited healthy DPs as a result of having a Mediterranean-like diet and lifestyle. A wide range of health benefits have been demonstrated for the Mediterranean diet and lifestyle, one of the most studied and well-known DPs in the world [72]. In the PREDIMED study (Prevention with Mediterranean Diet), high-risk patients who followed a Mediterranean lifestyle pattern exhibited inverse associations with hypertension, diabetes, obesity, and high cholesterol [73].

A diminished health status is associated with these variables (high energy-dense food intake and low levels of PA). To control sustained weight gain and maintain healthy lifestyles, nutrition education, health education, and nutritional counseling are recommended strategies [74]. It is important to mention that the Ministry of Public Health of Uruguay has published general recommendations for its population, but these are not expressed in daily servings, only in general messages. To better understand a healthy DP to follow, it may be helpful to convert those recommendations into numbers (daily servings).

Dietary principles of this diet include the consumption of fruits, vegetables, whole grain cereals, legumes, nuts and seeds, and fish, as well as the consumption of olive oil as the primary source of added fat. In addition to low dairy intake, namely yogurt and cheese, and low or moderate consumption of fish and poultry meat, moderate consumption of red meat, and modest wine consumption in adults, the Mediterranean diet also has certain other features [75,76].

According to prospective studies, adherence to the Mediterranean diet and lifestyle pattern reduces mortality, especially cardiovascular mortality, resulting in a longer life expectancy. Moreover, it has been associated with a reduced incidence of age-related cognitive dysfunction and neurodegenerative disorders, particularly Alzheimer’s disease [72].

Uruguay faces significant research challenges in an environment that has limited research capacity on PA-related indicators [77,78]. In national research, one of the major weaknesses is the lack of reliable data to enable epidemiological characterization [79]. Uruguay’s 2022 Report Card demonstrates a significant effort in this area [80]. In our study, there were no differences in PA levels between women and men from the UTE-study. In both gender groups, vigorous activity was the most common form of physical activity, based on METs/week. There was a difference between men and women in the amount of moderate and vigorous physical activity they engaged in, as well as the amount of time they spent sitting. Women spent less time sitting than men and engaged in less moderate or vigorous physical activity in general. Women spent a greater percentage of their time sitting than men.

The limitations of this study are related to the analysis itself. Studies that use PCA or cluster analysis consistently report subjective decisions as a weakness [13]. The majority of DPs identified, however, demonstrated significant reproducibility across different statistical solutions, fair relative validity, and high construct validity [14]. In addition, the limitations of this study relate to the self-reporting of important variables such as weight, height, and food consumption. Although the FFQ and IPAQ questionnaires have been validated, they are tools that provide an approximate representation of the subject’s actual condition. It is necessary to corroborate the findings of this type of study with clinical data. Strengths are determined by the sample size of the population. Considering the special nature of the IN-UTE study, the results could provide workplace nutrition and PA advice through outpatient clinics and ensure that the company has a health promotion policy in place, specifically related to nutrition and PA. Promoting the company on the internet would involve distributing useful content on the company’s website permanently. As a result of this population’s importance in Uruguay, follow-up activities are warranted.

## 5. Conclusions

In this study, dietary and PA clusters were investigated in the Uruguayan population. Among the four dietary clusters identified, energy-dense foods were consumed by all age groups, indicating an unhealthy DP. UTE healthier workers exhibited a more Mediterranean-like dietary and lifestyle pattern. Certain lifestyle and sociodemographic factors, including cumulative risk factors, night work, working outside the apartment, and moderate and intense PA, were significantly correlated with the dietary clusters, suggesting that socioeconomic status and PA may play an important role in determining these DPs to some extent. Accordingly, these findings can be used to design lifestyle interventions to reverse the appearance of unhealthy DPs in the UTE population based on the data obtained.

In light of our findings, regulatory authorities and professional organizations should take steps to reduce the consumption of energy-dense foods. It is necessary to conduct further research to determine how these and other variables influence the health status of workers and to assess the overall picture of the relationship between nutrients consumed and lifestyle factors in properly designed longitudinal studies involving the entire population. To establish the role of dietary and lifestyle factors as health determinants in Uruguayan populations, intervention studies focusing on DPs such as the Mediterranean diet and promoting PA may be necessary.

## Figures and Tables

**Table 1:** General description of the working population of Uruguay’s state-owned electric company (UTE).

*n*, percentage (%) and mean ± standard deviation			(Range)
Sociodemographic variables (n = 2194)			
Gender	Men	1393 (63.5)	
	Women	801 (36.5)	
Age (years)	Total	41.0 ± 10.9	(20–69)
	Men	40.4 ± 10.5	(21–69)
	Women	42.0 ± 11.4	(18–69)
Anthropometric variables (n = 2194); mean ± standard deviation			
Weight (kg)	Total	79.7 ± 17.1	(41–160)
	Men	85.5 ± 15.4	(47–160)
	Women	69.6 ± 15.2	(41–50)
Height (m)	Total	1.71 ± 0.09	(1.42–1.98)
	Men	1.76 ± 0.07	(1.54–1.98)
	Women	1.63 ± 0.06	(1.42–1.80)
BMI (kg/m^2^)	Total	27.1 ± 4.9	(16.3–52.1)
	Men	27.6 ± 4.5	(16.2–52.0)
	Women	26.2 ± 5.4	(16.5–51.9)
BMI Total categories, *n*, percentage (%)			kg/m^2^
Underweight		19 (0.9)	<18.5
Normal		782 (35.7)	18.5–<25
Overweight		918 (41.8)	25–<30
Obesity		475 (21.6)	>30
BMI Men categories, n, percentage (%)			kg/m^2^
Underweight		3 (0.2) *	<18.5
Normal		409 (29.4) *	18.5–<25
Overweight		653 (46.9) *	25–<30
Obesity		328 (23.5) *	>30
BMI Women categories, n, percentage (%)			kg/m^2^
Underweight		16 (1.9) *	<18.5
Normal		373 (46.6) *	18.5–<25
Overweight		265 (33.1) *	25–<30
Obesity		147 (18.4) *	>30
Characteristics of the job, n, percentage (%)			
On call 24 h a day (n = 2194)		273 (12.4)	
On call at night (n = 2194)		174 (7.9)	
Travels to another department (n = 2194)		388 (17.7)	
Schedule of rotations (n = 2194)		244 (11.1)	
At least one risk factor was present (n = 2194)		821 (37.4)	
Others		294 (13.5)	

* *p* < 0.05 comparison among gender BMI, body mass index.

**Table 2:** Eating behavior of employees of the Uruguayan state-owned electric company (UTE).

Food Groups		Daily Servings x ± SD	Grams/Day x ± SD	International Recommendations *
Cereals		3.0 ± 1.9	155.0 ± 98.0	170 g/d
Tubers		0.6 ± 0.5	59.0 ± 59.0	-
Legumes		0.1 ± 0.2	14.0 ± 25.0	-
Nuts		0.2 ± 0.4	8.0 ± 14.0	20 g/d
Milk and dairy products	Milk	0.6 ± 0.8	162.0 ± 195.0	720 g/d
	Yogurt	0.3 ± 0.7	44.0 ± 85.0	
	Cheese	0.4 ± 0.4	34.0 ± 33.0	
	Milk desserts	0.3 ± 0.5	34.0 ± 69.0	
Eggs		0.7 ± 0.8	31.0 ± 36.0	12 g/day
Meat and meat products	Meat, poultry, pork	1.3 ± 1.1	141.0 ± 120.0	105 g/d
	Beef and pork	0.8 ± 0.8	98.0 ± 100.0	
	Chicken	0.4 ± 0.5	44.0 ± 46.0	
	Meat products	0.9 ± 1.0	33.0 ± 32.0	
Fish and fish byproducts		0.2 ± 0.3	30.0 ± 37.0	32 g/d
Vegetables		1.1 ± 1.0	112.0 ± 100.0	375 g/d
Fruits and natural juices		2.1 ± 1.7	225.0 ± 189.0	250 g/d
	Fruits	1.7 ± 1.4	170.0 ± 140.0	250 g/d
Oils and fats	Oils	0.7 ± 0.8	7.0 ± 8.0	27 g/d
	Butter	0.2 ± 0.5	2.0 ± 5.0	
Sugar and sweets		1.6 ± 2.0	8.0 ± 10.0	-
Ultra-processed salted		0.8 ± 1.0	45 ± 42	-
Ultra-processed sweetened		1.3 ± 1.7	48 ± 62	-
Beverages	Water	5.4 ± 2.7	1078 ± 538	-
	Fruit nectars and soft drinks (sugar)	0.3 ± 0.8	60 ± 140	
	Fruit nectars and soft drinks (sweetener)	0.9 ± 1.5	99 ± 206	
	Alcohol	0.5 ± 0.8	69 ± 110	
Added salt		1.5 ± 1.7	3 ± 3	-
	Mealtime characteristics, n (percentage)			
Number of main meals per day		one		40 (1.8)
		two		214 (9.7)
		three		721 (32.9)
		four		1220 (55.6)
Number of main meals per day		Breakfast		1873 (85.3)
		Lunch		2149 (97.9)
		Snack		1647 (75.0)
		Dinner		1842 (84.0)
Meals between meals				1.4 ± 1.2

* Recommendations from Dietary Guidelines for Americans 2020–2025 [61,62] were used for the availability of these recommendations. To facilitate the comparison between grams per day (g/d) and ounce cups, these recommendations have been modified from ounces to grams as part of a healthy diet consisting of 2000 calories per day. x ± SD, mean ± standard deviation.

**Table 3:** Physical activity and sleep patterns of civil servants of the Uruguayan State Electric Company (UTE).

*n* (percentage)			Range
Sleep			
Total	<6 h	320 (14.6)	
	6 to 8 h	1838 (83.8)	
	9 to 12 h	36 (1.6)	
Men	<6 h	176 (12.8)	
	6 to 8 h	1189 (85.4) **	
	9 to 12 h	25 (1.8)	
Women	<6 h	141 (17.6)	
	6 to 8 h	648 (81.0) **	
	9 to 12 h	11 (1.4)	
Physical activity patterns (METs/weeks) mean ± standard deviation			
Total	Total	2382 ± 2453	
	Men	2654 ± 2674	
	Women	1911 ± 1930	
Light	Total	713 ± 849	
	Men	731 ± 903	
	Women	682 ± 745	
Moderate	Total	419 ± 692	
	Men	495 ± 751	
	Women	287 ± 553	
Vigorous	Total	1251 ± 1647	
	Men	1429 ± 1751	
	Women	941 ± 1396	

Mean ± standard deviation; ** *p* < 0.05 comparison between genders.

**Table 4:** Categorization of physical activity according to hours performed by employees of the state-owned electric company of Uruguay.

	Time Categories of Physical Activity				
		Sedentarism n (%)	Light n (%)	Moderate n (%)	Vigorous n (%)
Total	None	7 (0.3)	316 (14.4)	966 (44.0)	801 (36.5)
	<1 h	31 (1.4)	1238 (56.4)	631 (28.8)	469 (21.4)
	1–2 h	104 (4.7)	546 (24.9)	511 (23.3)	810 (36.9)
	2–3 h	209 (9.5)	61 (2.8)	64 (2.9)	91 (4.1)
	>3 h	1843 (84.0)	33 (1.5)	22 (1.0)	23 (1.0)
Men	None	6 (0.4)	207 (14.9)	522 (37.5)	428 (30.7)
	<1 h	27 (1.9) **	787 (56.5)	440 (31.6)	322 (23.1)
	1–2 h	88 (6.3) **	324 (23.3)	360 (25.8)	556 (39.9)
	2–3 h	181 (13.0) **	48 (3.4)	51 (3.7)	68 (4.9)
	>3 h	1091 (78.3) **	27 (1.9)	20 (1.4)	19 (1.4)
Women	None	1 (0.1)	109 (13.6)	444 (55.4) ***	373 (46.6) ***
	<1 h	4 (0.5) **	451 (56.3)	191 (23.8) ***	147 (18.4) ***
	1–2 h	16 (2.0) **	222 (27.7) **	151 (18.9) ***	254 (31.7) ***
	2–3 h	28 (3.5) **	13 (1.6) **	13 (1.6) ***	23 (2.9) ***
	>3 h	752 (93.9) **	6 (0.7) **	2 (0.2) ***	4 (0.5) ***
Time spent sitting (%)			Total	Men	Women
	None		7 (0.3)	6 (0.4)	1 (0.1)
	<1 h		31 (1.4)	27 (1.9) **	4 (0.5) **
	1–2 h		104 (4.7)	88 (6.3) **	16 (2.0) **
	2–3 h		209 (9.5)	181 (13.0) **	28 (3.5) **
	>3 h		1843 (84.0)	1091 (78.3) **	752 (93.9) **

Mean ± standard deviation; ** *p* < 0.05 comparison between genders; *** *p* < 0.001 comparison between genders.

## Data Availability

The raw data supporting the conclusions of this article will be made available by the authors, without undue reservation.

## Supplementary Materials

The following supporting information can be downloaded at https://www.mdpi.com/article/10.3390/nu16020304/s1, Table S1: Principal component analysis for workers in the IN-UTE study.
