# Supplementary material for: A Clustering Study of Dietary Patterns and Physical Activity among Workers of the Uruguayan State Electrical Company
Source: Nutrients. 2024 Jan 19;16(2):304. doi: 10.3390/nu16020304 (PMC10820101; doi:10.3390/nu16020304)
Supplement: Supplementary file 1 [file nutrients-16-00304-s001.zip › nutrients-2748566-supplementary.pdf]

Table S1. Principal component analysis of dietary patterns, physical activity, characteristics of the job and characteristics of the subject for workers in the IN-UTE study.

Variables	Energy-dense food DP	Characteristics of the job	Mediterranean-like DP	Subject's characteristics
Meat products	0.688			
Cereals	0.646			
Ultra-processed (salted and sugar)	0.64			
Beef meat	0.584			
Fruit nectars and soft drinks (sweetener)	0.552			
Milk and dairy products	0.435		0.25	
Beverages	0.331			0.299
Cumulative risk factors		0.923		
Work outside the apartment		0.737		
24-hour on-call duty		0.702		
Vegetables			0.686	
Fruits			0.675	
Legumes and Nuts			0.488	
Water			0.435	0.237
Oils and fats	0.334		0.374	
Vigorous physical activity			0.318	0.681
Moderate physical activity			0.258	0.565
Age			0.213	-0.559
Body mass index				-0.47
Eggs	0.213		0.3	0.419
Percentage of variance	12.862	10.979	9.521	7.325
Cumulative percentage of variance	12.862	23.841	33.363	40.688

Principal component analysis was used to maximize the information gained for the predominant food groups from diet. This mathematical model calculates new variables (principal components) that account for the variability in the food groups data and enables the study of covariances or correlations between variables (e.g., milk and dairy products, cereals, vegetables, meat and meat products, etc.). The combination of food group variables with the greatest amount of variability is the first principal component. The subsequent components (second and third principal components) describe the maximum amount of remaining variability. Factor loading was used to interpret the factor structure. Loadings are equivalent to Pearson correlation coefficients, and a higher loading indicates a stronger relation between a factor and an observed variable. Strong loading was defined as a value ≥0.6, and marginal loading as a value from 0.2 to 0.4.
